# Room-Temperature Perovskite Phase Transition of CsPbI_3_ for PV Manufacturing on Flexible Substrates

**DOI:** 10.1021/acsomega.4c10169

**Published:** 2025-02-13

**Authors:** Yifan Liu, Xuan Li, Levon Abelian, Chun Hei Lau, Zeyin Min, Yuying Hao, Stoichko Dimitrov

**Affiliations:** † College of Physics and Optoelectronics, Taiyuan University of Technology, Taiyuan 030024, China; ‡ School of Physical and Chemical Sciences, Queen Mary University of London, London E1 4NS, U.K.; § Helmholtz-Zentrum Berlin für Materialien und Energie GmbH, Hahn-Meitner-Platz 1, 14109 Berlin, Germany; ∥ College of Electronic Information and Optical Engineering, Taiyuan University of Technology, Taiyuan 030024, China

## Abstract

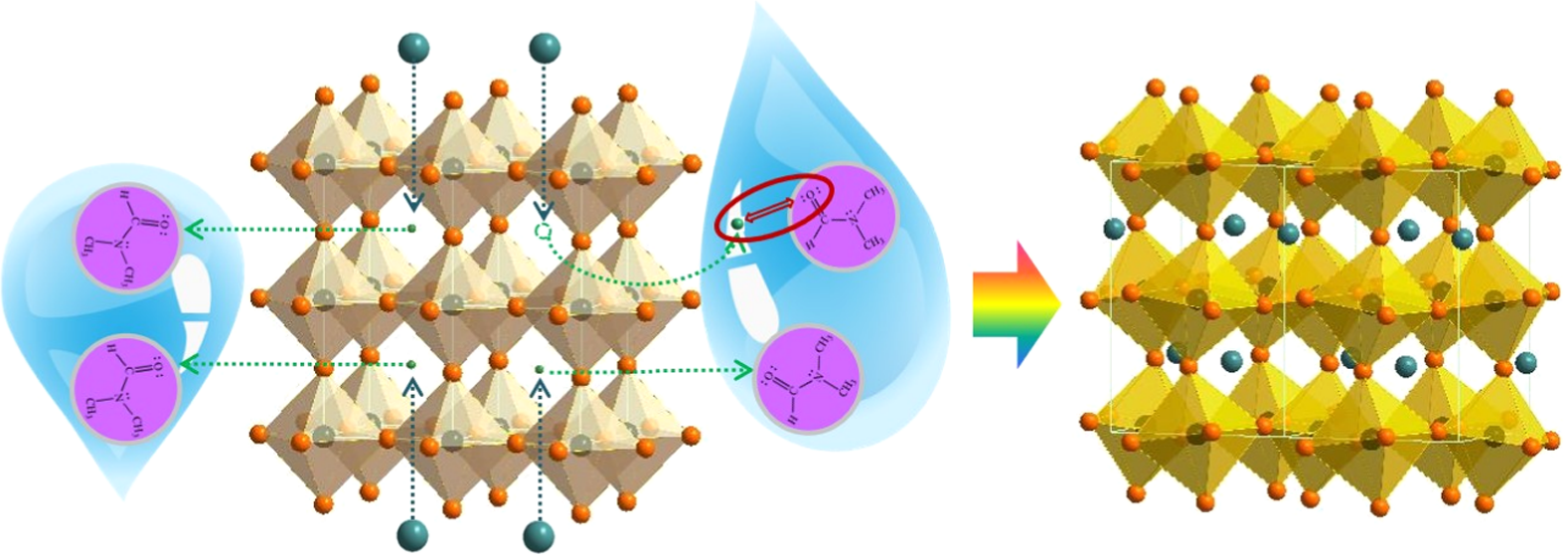

Printing perovskite films typically involves a high-temperature treatment exceeding 150 °C, which limits the manufacturing of flexible devices. All inorganic CsPbI_3_ perovskite is particularly promising for commercialization due to its high thermal stability. Herein, we discovered that when using DMF precursors containing CsI and HPbI_3_ for fabricating CsPbI_3_ films, an isopropanol (IPA) antisolvent bath immersion treatment of the wet films can enable a direct and rapid formation of optically active perovskite black phases at room temperature without annealing. In situ photoluminescence and in situ transmission techniques were employed to monitor and characterize the transition from the wet film to the final perovskite phase. It can be concluded that the relatively fast nucleation and slow grain growth during the IPA-bath treatment result in films with small grains and pronounced pinholes on the surface. Furthermore, FTIR, Raman, and NMR techniques were used to investigate changes in the chemical bonds. The characterization results revealed that the hydrogen in HPbI_3_ can form a chemical bond with the oxygen in DMF, resulting in mutual attraction. As DMF is extracted by IPA, the DMF molecule simultaneously induces the hydrogen to leave its original position, and then free cesium easily fills the vacancy left by hydrogen, forming the black-phase CsPbI_3_ perovskite. This finding reveals the mechanism of the room-temperature phase transition of CsPbI_3_ facilitated by IPA post-treatment, and it explains why the use of HPbI_3_ instead of PbI_2_ in the precursor solution effectively lowers the reaction energy barrier for CsPbI_3_ in previous works.

## 1 Introduction

**Figure 1:**
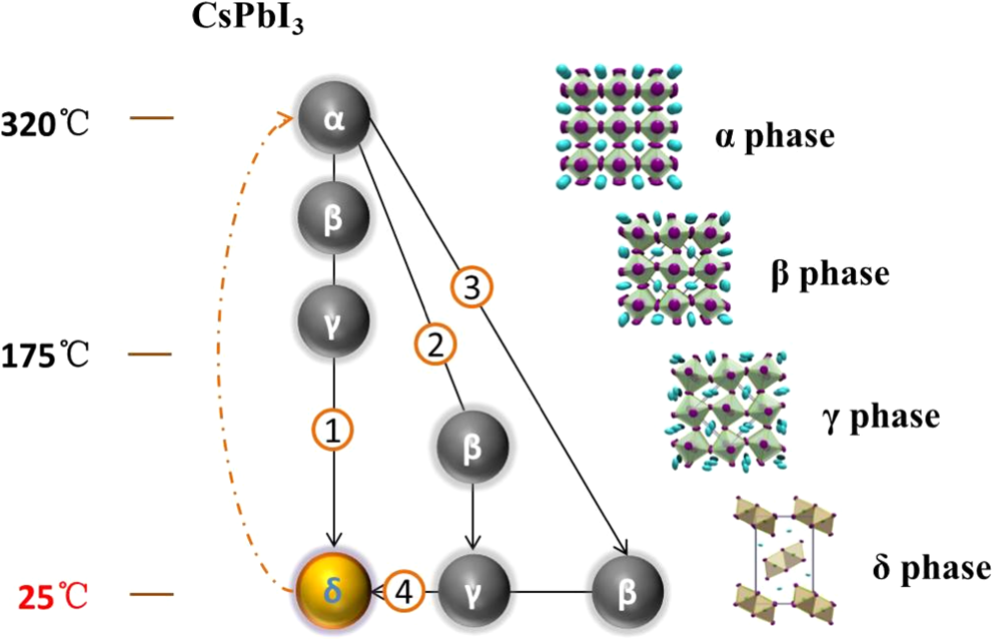
Phases of CsPbI_3_ along different temperature paths.

The inorganic lead halide perovskite CsPbI_3_ is a photoabsorbent material that exhibits excellent thermal and photoelectric stability and appropriate band gap (approximately 1.7 eV)^1−3^ for applications spanning across high-efficiency perovskite/silicon tandem solar cells, communication, aerospace, building-integrated photovoltaics, and automotive photovoltaic integration products.^4−6^ During fabrication, optically active black phases (α, β, γ phases) of CsPbI_3_ are only formed at high temperatures, while at room temperature (RT, 25 °C), the thermodynamically stable nonperovskite yellow phase (δ phase) is formed.^7,8^Figure 1 illustrates the phase transition process undergone by CsPbI_3_ along different temperature paths.^9^ Given the requirement for high temperature for phase transition to the black phases, current techniques for preparing CsPbX_3_ (X = Cl, Br, I) films typically involve high-temperature treatments exceeding 150 °C,^2,4,10−12^ as summarized in Table S1. However, commercially available flexible transparent conductive substrates like PET or PEN are not suitable for such temperatures, thus limiting the number of applications of this perovskite structure, especially for flexible devices.^13−15^

Based on the phase transition process of CsPbI_3_, it is theoretically possible to achieve room-temperature perovskite black phases via a preparation process at temperatures below 150 °C.^16,17^ Jiang et al. have shown that HPbX_3_ crystals can significantly reduce the crystallization energy barrier required for the formation of CsPbI_3_ black phases, thereby facilitating low-temperature phase transitions.^18^ Unfortunately, the underlying principles are not clear. Our own experience has shown that by substituting HPbI_3_ for PbI_2_ in the preparation of CsPbI_3_ films, the optimized annealing temperature can be reduced from 320 to 180 °C without altering the composition of the perovskite.^19,20^ Also, using HPbI_3_ as the source of both Pb and I can result in the preparation of more uniform and dense perovskite grains. Therefore, in many reported works, HPbI_3_ is preferred over PbI_2_ for the preparation of CsPbI_3_ perovskites.^10,21^

In addition to controlling the composition of perovskites, the choice of solvent also plays a crucial role in the phase transition temperature of perovskites.^22,23^ Wang et al. addressed the issue of slow crystallization of CsPbIBr_2_, caused by the high boiling point of dimethyl sulfoxide (DMSO), by introducing low-boiling-point alcohol solvents such as methanol and ethanol to form a low-boiling-point DMSO-alcohol solvent mixture.^16^ This approach effectively reduces the reaction energy barrier and accelerates the crystallization of the CsPbX_3_ all-inorganic perovskites.

**Figure 2:**
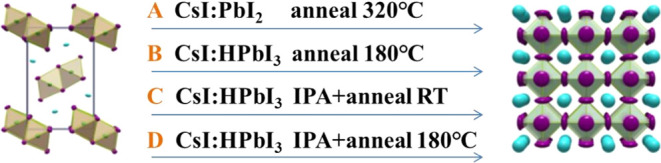
Four approaches for solution-based preparation of CsPbI_3_ perovskite.

In this study, we report the discovery of a novel antisolvent bath immersion treatment using isopropanol (IPA) that enables the direct and rapid formation of optically active black-phase CsPbI_3_ perovskite at room temperature. Four approaches for preparing CsPbI_3_ perovskites were studied, as summarized in Figure 2. Unlike existing solvent-assisted methods that primarily focus on small-area films (<1 cm^2^) or rely on post-treatment techniques such as solvent dripping or controlled solvent annealing atmospheres,^2,17,24^ our approach is designed specifically for scalable and low-cost fabrication of flexible large-area films. By immersing blade-coated wet films in an IPA antisolvent bath, we have demonstrated a simple yet highly effective method for processing all-inorganic perovskite films at low temperatures, overcoming key challenges associated with traditional spin-coating-based methods and high equipment requirements.

Moreover, the mechanism of antisolvent action in this work fundamentally differs from previously reported methods.^16,25^ We show that hydrogen in HPbI_3_ interacts with oxygen in DMF, creating mutual attraction. During immersion in the IPA antisolvent bath, DMF is extracted by IPA, inducing the hydrogen to vacate its original position and allowing free cesium ions to occupy these vacancies. This effectively lowers the reaction energy barrier and facilitates the direct formation of black-phase CsPbI_3_ perovskites at room temperature, as supported by in situ optical spectroscopic analysis, NMR, and FTIR data. By providing a scalable, low-temperature, and low-cost method for fabricating high-quality CsPbI_3_ films, this work paves the way for the advancement of perovskite-based technologies.

## 2 Experimental Section

### 2.1 Materials

PbI_2_ was purchased from Merck (UK). HPbI_3_ (99%) was purchased from Xi’an Polymer Light Technology. CsI and *N*,*N*-dimethylformamide (DMF) were obtained from Thermo Fisher Scientific. 2-Propanol (IPA) was obtained from Honeywell.

### 2.2 Preparation of the CsPbI_3_ Film

One hundred and fifty-six milligrams of CsI and 353.4 mg of HPbI_3_ were dissolved in 1 mL of DMF solvent. The molar ratio of CsI to HPbI_3_ is 1:1, yielding a precursor solution of CsPbI_3_ with a concentration of 0.6 M. The prepared precursor solution was stirred using a magnetic stirrer for 6 h and then filtered using a 0.22 μm pore size filter. The precursor of CsI and PbI_2_ with a concentration of 0.6 M was prepared by following a similar procedure. The wet film of perovskite was manually blade-coated onto a glass substrate using a surgical blade. The wet film was then immersed in IPA solution for 2 min. Subsequently, the wet film was either left to stand by or subjected to further heating at different temperatures (RT–180 °C) for 10 min in the glovebox before characterization tests.

### 2.3 Characterization

In-situ transmittance and photoluminescence (PL) spectra were performed by Avantes spectrometer and fibers, with an incident laser beam at 405 nm. Atomic force microscopy (AFM) images were obtained from the dimension icon scanning probe microscope (Bruker). Raman spectra were collected by using a Renishaw inVia Raman microscope operated with an incident laser beam at 442 nm. For the nuclear magnetic resonance (NMR) results, ^1^H NMR spectroscopies were performed on a Bruker HD 400 MHz spectrometer. Also, an FEI Inspect scanning electron microscope (SEM), Siemens D5000 X-ray Powder diffractometer (XRD), Krüss drop shape analyzer, and Shimadzu IRTracer-100 Fourier transform infrared spectrophotometer (FTIR).

### 2.4 Selection of Antisolvent

Thirteen different antisolvents were tested for the antisolvent immersion method, including DEE (diethyl ether), EA (ethyl acetate), IPA (isopropanol), EtOH (ethanol), methanol, XYL (m-xylene), Tol (toluene), Mesit (mesitylene), DCB (1,2-dichlorobenzene), DIE (diisopropyl ether), Ani (anisole), CB (chlorobenzene), and CF (chloroform). Among these, IPA, EtOH, and methanol successfully induced a color change in the perovskite wet films from yellow to black at room temperature. However, it was observed that films treated with EtOH and methanol still required annealing above 180 °C to achieve relatively stable films, whereas IPA-treated films did not require annealing to attain comparable stability. Therefore, this study focuses on IPA as the antisolvent of choice.

## 3 Results and Discussion

### 3.1 Surface

**Figure 3:**
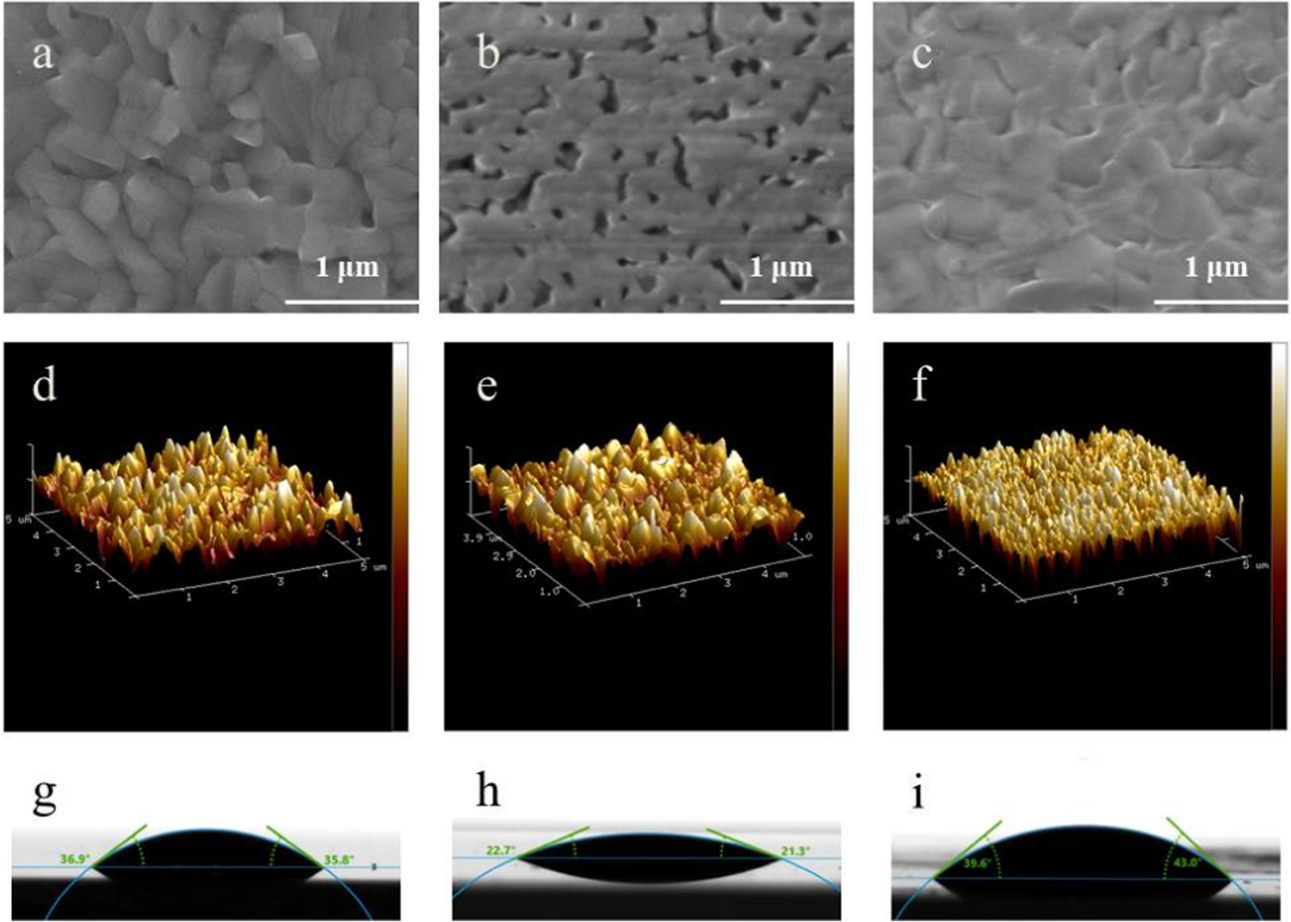
SEM results of (a) control, (b) IPA-RT, and (c) IPA-180C samples; AFM images of (d) control, (e) IPA-RT, and (f) IPA-180C samples. Water contact angle test results of (g) control, (h) IPA-RT, and (i) IPA-180C samples.

Films were blade coated from a DMF precursor solution containing CsI and HPbI_3_ and treated via approaches B, C, and D, described in Figure 2. Approach B included annealing of the wet film at 180 °C and was used as the control experiment. Approach C included an IPA-bath treatment at room temperature, noted as IPA-RT. Approach D included an IPA-bath treatment of the wet film followed by thermal annealing at 180 °C, noted as IPA-180C. The surface morphology of the final films was analyzed using SEM, Figure 3a–c. The grains in all of the samples were relatively uniform, with average grain sizes of 330, 280, and 320 nm for control, IPA-RT, and IPA-180C, respectively. The IPA-RT sample (Figure 3b) exhibited more prominent pinholes at the microscale. However, the IPA-180C sample (Figure 3c) demonstrated that after annealing at 180 °C, the pinholes became significantly less noticeable. We hypothesize that the appearance of pinholes in IPA-RT is most likely due to the slower growth in the IPA solvent bath treatment, resulting in smaller grain sizes; this will be further discussed in this paper based on in situ spectral characterization. When the IPA-RT sample is annealed at 180 °C, according to the Ostwald ripening principle, the grains can grow larger, and the gaps between grains decrease; hence, the pinholes vanish.^26^

AFM images of these three samples are presented in Figure 3d–f. The average surface roughness (*R*_a_) of the IPA-RT sample was approximately 35.6 nm (Figure 3e), significantly higher than the control’s 16.4 nm (Figure 3d) and IPA-180C’s 11.0 nm (Figure 3f). This result was consistent with the SEM findings, as the surface of the IPA-RT sample exhibited obvious pinholes. While, in comparison to the control, IPA-180C demonstrated lower surface roughness, indicating that IPA-bath treatment can effectively enhance the surface smoothness of the sample. The water contact angle test results are presented in Figure 3g–i. The angles of the control, IPA-RT, and IPA-180C samples were 36.3, 22.0, and 41.3°, respectively. These results were also consistent with the SEM and AFM findings. Due to surface pinholes, IPA-RT exhibited increased hydrophilicity, with a water contact angle close to that of a pure glass surface (approximately 20.9°, see Figure S1). In comparison to the control, IPA-180C was more hydrophobic due to its smoother surface. Regarding film stability, experiments indicated that the stability of CsPbI_3_ films treated with IPA immersion is highly dependent on film quality and ambient humidity, which aligns with previously reported studies demonstrating that CsPbI_3_ films exhibit excellent stability under relative humidity below 30%.^19,20,27^

### 3.2 In Situ Spectrum

**Figure 4:**
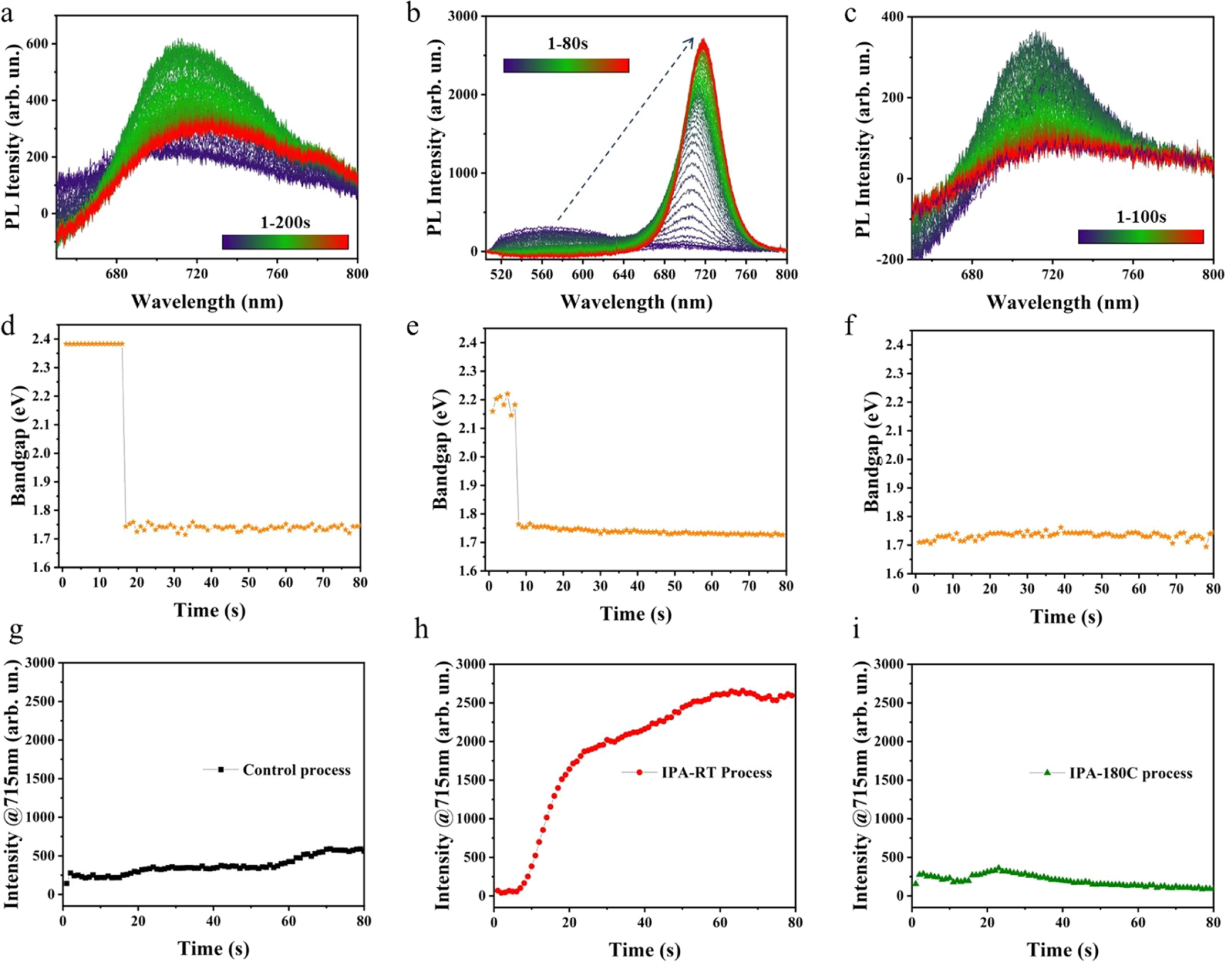
In situ PL spectra of (a) the control process, (b) IPA-RT process, and (c) IPA-180C process; fitted band gap changes of (d) the control process, (e) IPA-RT process, and (f) IPA-180C process; PL intensity changes at 715 nm of the (g) control process, (h) IPA-RT process, and (i) IPA-180C process.

**Figure 5:**
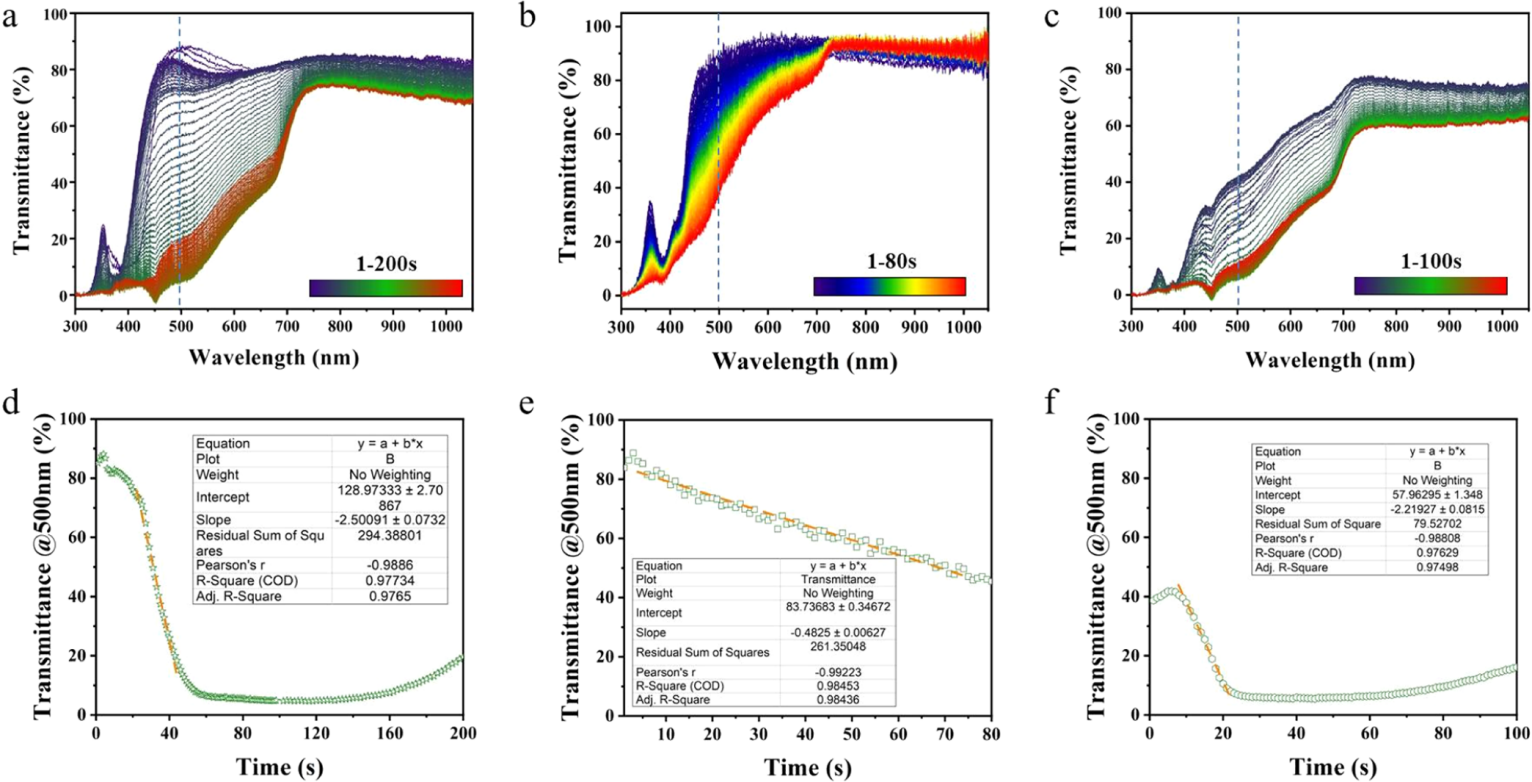
In situ transmittance results of the (a) control process, (b) IPA-RT process, and (c) IPA-180C process; overall transmittance trend changes at 500 nm of the (d) control process, (e) IPA-RT process, and (f) IPA-180C process; (d, e, f) inset tables show the fitted results of the transmittance change rate during the grain growth stage.

To understand the process of perovskite crystal growth, processes B, C, and D were monitored through in situ photoluminescence (PL) (Laser 405 nm) and in situ transmission (spectral range 300–1100 nm). For the control sample, the wet film was annealed at 180 °C for 200 s. For IPA-RT, the wet film was kept in IPA for 80 s. For IPA-180C, after 80 s of IPA-bath treatment, annealing was performed at 180 °C for 100 s. The corresponding results are presented in Figures 4 and 5.

The in situ PL data can give signals when crystals are formed. From the control process (Figure 4a), the position of the PL peak undergoes a shift from around 520 nm to 710 nm after approximately 16 s of annealing, followed by a slight redshift. This change is highly probable when the back-phase crystals are formed. By estimating the change in band gap over time using 1240 divided by the peak wavelength (Figure 4d), the band gap of the wet film is 2.38 eV, a value close to that of the δ-phase CsPbI_3_ (2.9 eV). Furthermore, the wet film appears pale yellow after standing, consistent with the yellow color of the δ-phase. Therefore, we infer that the main component in the wet film is the δ-phase CsPbI_3_. The possibility that the wet film mainly consists of HPbI_3_ perovskite is ruled out because the PL results of HPbI_3_ (Figure S2) show a peak at approximately 735 nm (band gap 1.68 eV). After annealing the wet film for 16 s, the band gap abruptly shifts to 1.74 eV and stabilizes at around 1.73 eV, indicating the formation of the black-phase perovskite. Based on our previous studies of this control process, the perovskite formed is γ-CsPbI_3_.^19^

The in situ PL results of the IPA-RT process (Figure 4b) show that the position of the PL peak shifts from around 560 nm initially to 707 nm after approximately 7 s of antisolvent treatment time, followed by a gradual weak redshift and finally stabilizes at around 716 nm. The corresponding changes in the band gap are illustrated in Figure 4e. This trend generally aligns with the in situ PL trend of the control process. Additionally, during the IPA-bath treatment process, a noticeable color change from yellow to dark brown can be observed. As shown in Figure 4e, the band gap abruptly shifts from 2.21 eV (yellow phase) to 1.75 eV (black phase), followed by gradual stabilization at approximately 1.73 eV. Compared to the initial peak position of the control process (520 nm), one of the IPA-bath processes (560 nm) shifts closer to the infrared spectral region. This suggests that the phase transition process begins as soon as the wet film contacts IPA, and after 7 s, the phase transition process of the entire film is mostly completed. Therefore, the band gap value detected initially is closer to the value of the black-phase perovskite.

The in situ PL spectra and corresponding band gap changes of the IPA-180C process are presented in Figure 4c,f, respectively. It can be observed that after IPA-bath treatment, there is no significant shift in the PL peak position during the subsequent heating process, with minor fluctuations around approximately 715 nm, corresponding to a band gap value of 1.73 eV. Combining both the IPA-RT and IPA-180C stages indicates that IPA-bath treatment can rapidly convert the perovskite from the yellow phase to the black phase, and the subsequent annealing process does not significantly affect the perovskite’s phase.

In the in situ PL spectra here, a negative signal can be observed, which is mainly caused by the misalignment of the detection fiber. In Figure 4a,c, the negative PL signal is more pronounced, and the overall PL intensity is lower due to the need for continuous heating of the sample on a hot plate during the detection process. Additionally, in all three processes, the PL intensity gradually increases at the beginning. We believe that the PL intensity increase is due to the continuous formation of the black phase. Figure 4g–i shows the change in PL intensity at 715 nm over time for the control, IPA-RT, and IPA-180C processes. The 715 nm wavelength was chosen because the PL intensity changes significantly at this point. It can be observed that the rate of change for the IPA-RT process is significantly higher than that of the control and IPA-180C processes, especially within 20 s, indicating that the nucleation rate of the black phase during the IPA treatment is much higher than in the other two processes. And a decrease in PL intensity indicates the onset of sample degradation.

To double-check the phase of CsPbI_3_ after IPA treatment, we subjected samples to a series of annealing temperatures from room temperature to 180 °C. As the annealing temperature increased, the color of the black film samples gradually darkened (see Figure S3). XRD results (see Figure S4) show that the final samples are similar, with the peaks at ∼12° corresponding to the (012) crystal plane of γ-CsPbI_3_.^28^ There is a slight tendency for the XRD peak positions to shift toward larger angles, indicating a slight decrease in the lattice constants of the perovskite crystals, which implies an increase in symmetry. This trend aligns with the material properties of CsPbI_3_, which theoretically exists in a highly symmetric cubic phase (α-phase) at 320 °C.

The in situ transmittance results of the control process are illustrated in Figure 5a. When the effects of reflection and scattering are ignored, the reduction in transmission can be attributed to the enhancement of absorption, which can be used as an indicator for the crystal grain growth. Spectral analysis reveals significant variations in the transmission of visible light (400–780 nm) and of ultraviolet light at ∼350 nm, but the changes in the near-infrared range (800–1000 nm) are relatively minor. The spectra are consistent with the optical properties of CsPbI_3_ and confirm the formation of the perovskite material.

According to Figure 5a, the transmittance changes during annealing of the control sample are more gradual than the sudden changes observed in in situ PL, but within the same time frame. Before 20 s, the transmittance has an increase and then a slow decrease. This period is considered as the nucleation of the black phase as a time frame required for the phase transition observed in the in situ PL results (16 s). This is better seen in the transmittance data at 500 nm in Figure 5d. From 20 to 50 s, the transmittance decreases rapidly. The rate of change is ∼2.5%/s, as obtained through linear fitting (Figure 5d inset). We link this process to crystal growth. Between 50 and 150 s, the transmittance remains relatively stable, indicating that the size of the grains no longer changes significantly. After 150 s, signs of decomposition start to appear in the sample due to heating and exposure to probing light, leading to a slight increase in transmittance.

The in situ transmittance results of the IPA-RT process are depicted in Figure 5b. The transmittance change distributions in the ultraviolet, visible, and infrared range are similar to those in the control process. However, when analyzing the trend of the entire process (1–80 s) using transmittance data at 500 nm (Figure 5e) as an example, significant differences are evident. The in situ PL results above indicate that the transition to the black phase only takes approximately 7 s during the IPA-RT process. Here, we observe a slight increase in transmittance within 4 s, followed by a gradual decrease in transmittance at a rate of approximately 0.48%/s (Figure 5e inset) until around 75 s, where it stabilizes. These PL and transmittance results mutually support each other, suggesting that during the IPA-RT process, the wet film undergoes a rapid transition to the black phase within a very short time frame (approximately 4–7 s), which is about 4 times faster than in the control process. Thereafter, in the antisolvent, the growth rate of the grains is relatively slow, approximately 1/5 of that under high-temperature annealing conditions. Combining with the previous SEM results (Figure 3b), it can be reasonably speculated that the relatively faster nucleation and slower grain growth during IPA-bath treatment will dominantly result in the film samples with a final grain size of approximately 280 nm, with very pronounced pinholes on the surface.

Figure 5c illustrates the changes in transmittance of the film samples during the IPA-180C annealing process within 100 s. The starting position here is roughly consistent with the ending position in the IPA-RT process, while the final position is consistent with that of the control process. The transmittance at 500 nm (Figure 5f) begins to decrease after 7 s of heating with a rate of ∼2.2%/s (Figure 5f inset). This indicates that after the sample reaches a stable state under 180 °C annealing conditions, most likely according to the Ostwald ripening principle, the grains can continue to grow. The growth rate of the grains and the final grain size here are comparable to those of the control process. After 20 s, the transmittance stabilizes, and the gradual increase in transmittance after 70 s suggests that the sample possibly begins to decompose.

### 3.3 Solution and Chemical Bond

After the in situ transition process of IPA-treated CsPbI_3_ wet films is discussed, it is crucial to explore the chemical changes occurring during the phase transition. As shown in Figure S5, the solubilities of 0.6 mmol of CsI and HPbI_3_, as well as their respective solubilities in 1 mL of DMF, IPA, and a DMF and IPA mixed solution, are presented. The original solution (OR) refers to the liquid after shaking and stirring, while the filtered solution (FI) refers to the clear liquid after filtration using a 0.22 μm filter. The intuitive results of the solubility and solution color for the OR and FI series show that CsI is soluble in DMF, insoluble in IPA, and sparingly soluble in the DMF and IPA mixed solution. HPbI_3_ is soluble in DMF, insoluble in IPA, and sparingly soluble in DMF and IPA mixed solution. The mixture of CsI and HPbI_3_ is completely soluble in DMF, is insoluble in IPA, and quickly precipitates yellow-brown sediment in the DMF and IPA mixed solution. The solubility test also further confirms that the IPA antisolvent can rapidly extract the DMF solution from the CsPbI_3_ wet film, thereby promoting the formation of the black-phase perovskite.

To investigate the changes in chemical bonds within the solutions, FTIR was conducted in transmission mode for the OR and FI series (Figure S6). As shown in Figure S6a,c, when CsI is added to DMF, IPA, and the DMF and IPA mixed solution, the main positions of the transmission peaks remain unchanged for both the original and filtered solutions. However, when HPbI_3_ is added to the DMF solution (Figure S6b), a weak transmission peak appears at 1020 cm^–1^, indicating a chemical interaction between HPbI_3_ and DMF. This position is most likely associated with the C–O stretching of a C–O–H group formed from C=O in DMF and H in HPbI_3_. Similar results were also detected in the FI series, as shown in Figure S6d.

**Figure 6:**
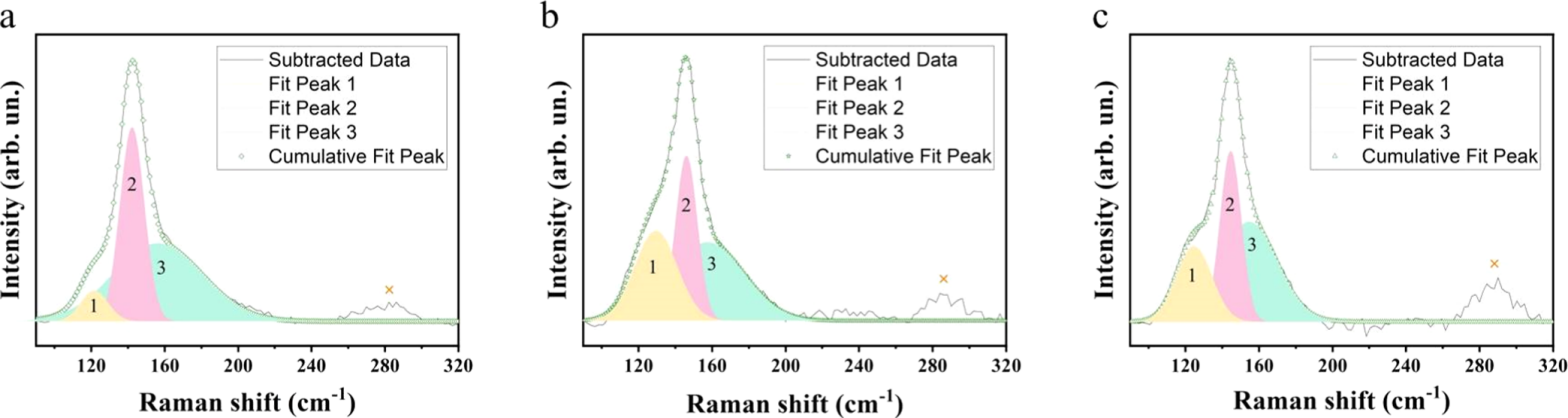
Experimental Raman spectrum and its peak fitting results of the δ-CsPbI_3_ film samples of (a) Control, (b) IPA-RT, and (c) IPA-180C.

The chemical bonds in the solid films were examined by Raman spectroscopy. Previous reports indicated that vibrational Raman modes are not allowed in cubic α-CsPbI_3_ for symmetry reasons.^29^ Our experimental Raman spectrum collected on the black α-phase sample did not show any Raman features. It was also observed that the samples degraded to the δ-yellow phase during exposure to the strong Raman laser. Figure 6a–c shows the Raman spectra of the δ-CsPbI_3_ film samples of Control, IPA-RT, and IPA-180C. Curve fitting was used to extract peak positions. Due to the notch filter, peaks at frequencies smaller than 90 cm^–1^ could not be observed. Between 90 and 200 cm^–1^, the spectra of the samples were similar, with three peaks for each. The peaks for the control sample were at 121, 142, and 155 cm^–1^; for the IPA-RT film at 129, 146, and 157 cm^–1^; and for the IPA-180C film at 124, 144, and 154 cm^–1^. According to reported studies, the peaks above 80 cm^–1^ mainly concern vibrations of lead and iodide atoms.^29^ The slight differences in the positions of fitted peaks indicate some variation in the vibrational modes of lead and iodide atoms among the different samples. Additionally, all three samples exhibit a peak at around 285 cm^–1^, which again reveals the presence of the CsPbI_3_ δ-phase.

**Figure 7:**
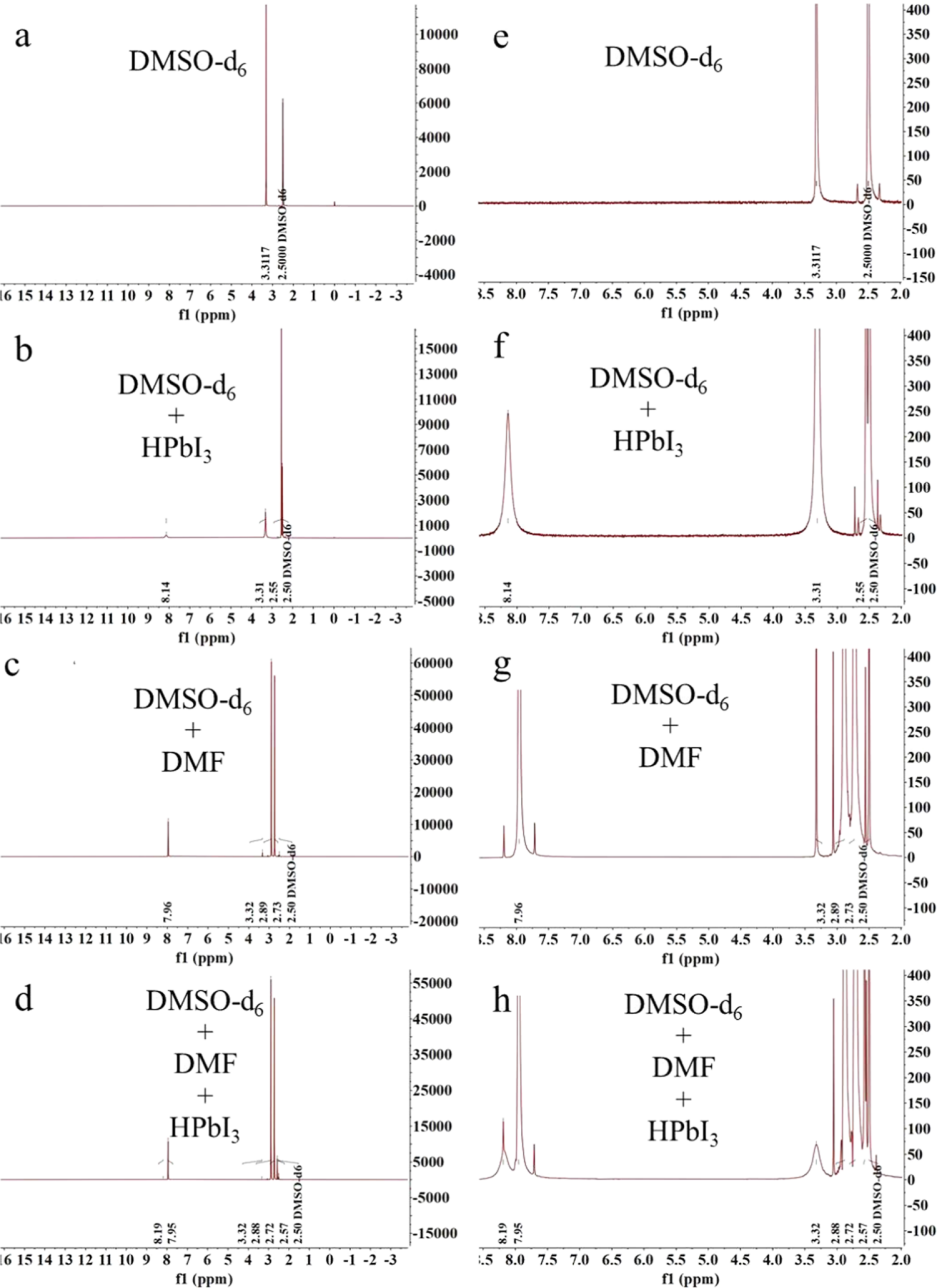
Complete ^1^H NMR spectra of (a) DMSO-*d*_6_, (b) DMSO-*d*_6_ solution with dissolved HPbI_3_ powder, (c) DMSO-*d*_6_ solution with dissolved DMF, and (d) DMSO-*d*_6_ solution with dissolved HPbI_3_ powder and DMF. Enlarged details of the ^1^H NMR spectra of (e) DMSO-*d*_6_, (f) DMSO-*d*_6_ + HPbI_3_, (g) DMSO-*d*_6_ + DMF, and (h) DMSO-*d*_6_ + DMF + HPbI_3_.

To further verify the possible chemical bond interactions in the process of forming black CsPbI_3_ films, we measured the proton nuclear magnetic resonance (^1^H NMR) spectrum of DMF and HPbI_3_ dissolved in dimethyl sulfoxide-d_6_ (DMSO-*d*_6_). Four samples were tested: pure DMSO-*d*_6_, DMSO-*d*_6_ solution with dissolved HPbI_3_ powder, DMSO-*d*_6_ solution with dissolved DMF, and DMSO-*d*_6_ solution with dissolved HPbI_3_ powder and DMF. The complete spectra of the four samples are shown in Figure 7a–d, with detailed spectra in Figure 7e–h.

As shown in Figure 7a,e (DMSO-*d*_6_), the signal of DMSO-*d*_6_ is located at 2.50 ppm with a pair of symmetric ghost peaks on either side. The peak at 3.31 ppm corresponds to the signal of H_2_O. In Figure 7b,f (DMSO-*d*_6_ + HPbI_3_), besides the signals at 2.50 ppm (DMSO-*d*_6_) and 3.31 ppm (H_2_O), there is a signal at 2.55 ppm, which corresponds to the H–I bond from HPbI_3_, also with symmetric ghost peaks. The signal at 8.14 ppm likely corresponds to an H–O bond, or possibly an H–S bond, due to the presence of uncoordinated electron pairs on both O and S in DMSO (Figure S7).^30^

In Figure 7c,g (DMSO-*d*_6_ + DMF), the 2.50 ppm peak is from DMSO-*d*_6_, and the 3.32 ppm peak is from H_2_O. The peaks at 7.96, 2.89, and 2.73 ppm all correspond to DMF. In Figure 7d,h (DMSO-*d*_6_ + DMF + HPbI_3_), the 2.50 ppm signal is from DMSO-*d*_6_, the 3.32 ppm signal is from H_2_O, and the 2.57 ppm signal is from the H–I bond. The peaks at 7.95, 2.88, and 2.75 ppm correspond to DMF. Notably, a distinct peak at 8.19 ppm appears, which is most likely due to an H–O bond.^30^ Considering that HPbI_3_ can provide a proton and DMF contains uncoordinated electron pairs on O (Figure S7), this signal is highly plausible.

It is important to note that by comparing the spectra, both DMSO and DMF have uncoordinated electron pairs on the atom of O, and when HPbI_3_ provides a proton in the solution, NMR detects signals at 8.14 ppm (Figure 7f) and 8.19 ppm (Figure 7h), which are very close in position. Therefore, we are inclined to believe that the peaks at 8.14 ppm in Figure 7f and 8.19 ppm in Figure 7h both correspond to the H–O bond. This result further confirms the reliability of the NMR analysis for the results shown here. Additionally, the NMR analysis results here are also consistent with the above FTIR analysis results, which indicate a chemical interaction between HPbI_3_ and DMF, most likely originating from stretching of the C–O–H mode.

### 3.4 Mechanism

**Figure 8:**
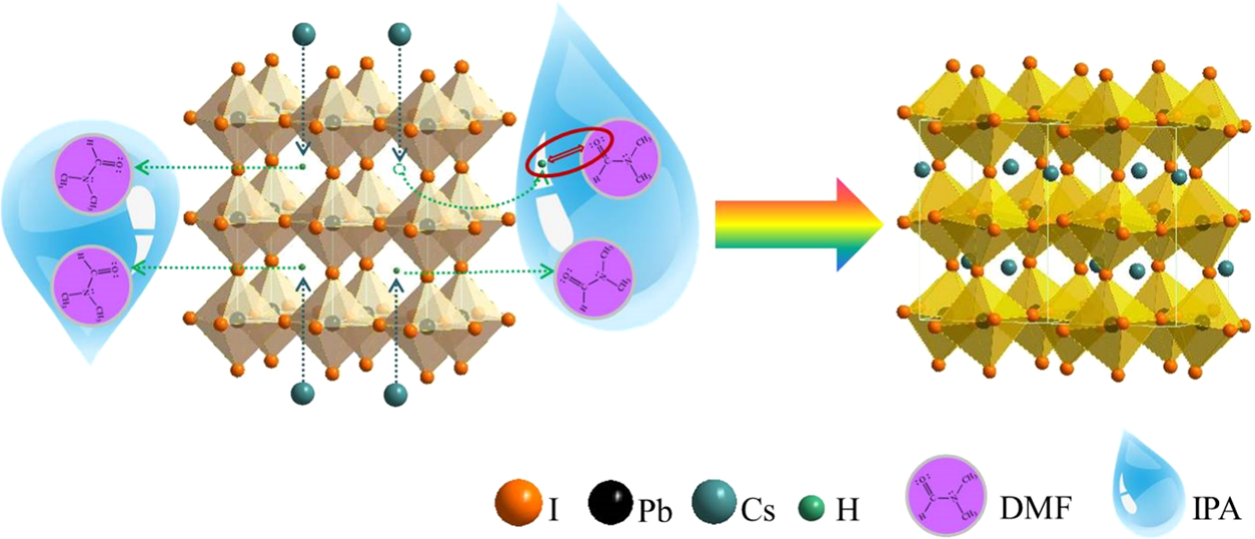
Diagram of the mechanism for the CsPbI_3_ room-temperature phase transition facilitated by IPA-bath treatment.

Based on the spectroscopic analysis in this paper, the mechanism for the room-temperature phase transition of CsPbI_3_ facilitated by IPA post-treatment is proposed (Figure 8). HPbI_3_ possesses an ABX_3_ structure, where the H at the A site can form a chemical bond with the oxygen in DMF, which contains lone pair electrons, resulting in mutual attraction. When IPA is used as an antisolvent to treat the wet film, IPA can quickly extract DMF but cannot dissolve the perovskite material. As DMF is extracted, it simultaneously induces H in the HPbI_3_ structure to leave its original position. The free Cs then fills the vacancy left by H to maintain structural stability, forming an optically active black-phase CsPbI_3_ perovskite. Considering that the radius of Cs is much larger than that of H, the substitution action stabilizes the ABX_3_ structure further. Thermodynamically, this Cs substitution for H is easily facilitated, which ultimately leads to the black-phase transition occurring at low temperatures, such as room temperature.

This mechanism also explains why the black-phase perovskite obtained from a DMF precursor solution of CsI and PbI_2_ requires annealing at 320 °C, whereas the use of HPbI_3_ instead of PbI_2_ reduces the annealing temperature for the black phase to 180 °C. It is mainly because the H in HPbI_3_ forms a bond with the oxygen in DMF, and as DMF evaporates, it induces the H to leave its original position, significantly lowering the energy barrier for Cs to replace H, thus resulting in a lower phase transition temperature.

## 4 Conclusions

In this paper, we presented our finding that when using DMF precursors containing CsI and HPbI_3_ for fabricating CsPbI_3_ films, an isopropanol (IPA) antisolvent bath immersion treatment of wet printed films can enable a direct and rapid formation of optically active perovskite black phases at room temperature without annealing. Through a comparative study of the film surface morphology, we observed that the CsPbI_3_ samples exhibited pinholes on the microscale. However, after annealing at 180 °C, the pinholes became significantly less noticeable.

To elucidate the perovskite phase transition process, we monitored the transition from the wet printed film to the final perovskite film using in situ photoluminescence and transmission techniques. The results indicated that the relatively fast nucleation and slow grain growth during the IPA-bath treatment predominantly resulted in film samples with pinholes on the surface. After the sample reaches a stable state under 180 °C annealing conditions, highly likely following the Ostwald ripening principle, the pinholes disappear.

FTIR and NMR analysis revealed that when HPbI_3_ is added to the DMF solution, a chemical interaction occurs between HPbI_3_ and DMF, involving the bonding of the hydrogen from HPbI_3_ and the oxygen from DMF. It was concluded that when IPA is used as an antisolvent, it can quickly extract DMF from the printed film and induce the H from HPbI_3_ to also leave its position. Cesium then fills the H-vacancy to maintain structural stability, forming an optically active black-phase CsPbI_3_ perovskite. This explains why the use of HPbI_3_ as a raw material can lower the reaction energy barrier for forming black-phase CsPbI_3_ in previous works.

This work therefore reports a new method for CsPbI_3_ synthesis at low temperatures using an antisolvent bathing approach that can benefit manufacturing by lowering energy usage and enabling printing on flexible plastic substrates. It also provides an explanation for the underlying mechanism of this process. Future work should investigate the thermodynamic energy changes during the reaction process, as understanding these could lead to more efficient and optimized fabrication methods. Considering the potential energy savings and investment reductions that could result from scaling this method for photovoltaic manufacturing, this represents a valuable direction for research and development.

## Supporting Information Available

Summary of performance, structure, and fabrication methods of published all-inorganic CsPbX_3_ solar cells; water contact angle measurements; photoluminescence of HPbI_3_; Images, XRD, solubility, and FTIR of CsPbI_3_ samples. (PDF)
