# Supplementary material for: Room-Temperature Perovskite Phase Transition of CsPbI3 for PV Manufacturing on Flexible Substrates
Source: ACS Omega. 2025 Feb 13;10(7):7102–11. doi: 10.1021/acsomega.4c10169 (PMC11865993; doi:10.1021/acsomega.4c10169)
Supplement: Supplementary file 1 — ao4c10169_si_001.pdf [file ao4c10169_si_001.pdf]

Supporting Information

Room-Temperature Perovskite Phase Transition of $CsPbI_3$ for PV Manufacturing on Flexible Substrates

Yifan Liu[a,b], Xuan Li[c], Levon Abelian[b], Chun Hei Lau[b], Zeyin Min[b], Yuying Hao[d]*, Stoichko Dimitrov [b]*

a. College of Physics and Optoelectronics, Taiyuan University of Technology, Taiyuan 030024, China
b. School of Physical and Chemical Sciences, Queen Mary University of London, London E14NS, UK
c. Helmholtz-Zentrum Berlin für Materialien und Energie GmbH, Hahn-Meitner-Platz 1, 14109 Berlin, Germany
d. College of Electronic Information and Optical Engineering, Taiyuan University of Technology, Taiyuan 030024, China

*Corresponding author:
Stoichko Dimitrov
E-mail: s.dimitrov@qmul.ac.uk
Yuying Hao
E-mail: haoyuying@tuyt.edu.cn

Table of Contents

Content Pages

Table S1. Summary of all-inorganic $CsPbX_3$ solar cells……………………………3

Figure S1. The water contact angle results of a pure glass surface………………4

Figure S2. The photoluminescence (PL) results of $HPbI_3$ powder………………..5

Figure S3. Photos of $CsPbI_3$ film samples………………………………………...6

Figure S4. XRD results of $CsPbI_3$ samples……………………………………….7

Figure S5. Solubility test visual photos……………………………………………8

Figure S6. FTIR results in transmission mode……………………………………9

Figure S7. Chemical molecular structures of DMF and DMSO…………………10

References…………………………………………………………………………11

Table S1

Summary of all-inorganic $CsPbX_3$ solar cells with areas larger than 0.1 cm².

	Reference	Year	Solar cell structure	Active area / cm^2	PCE	Perovsktie	
						Fabricating Method	Treatment Temperature
$CsPbI_3$	1	2017	FTO/c-TiO_2/$CsPbI_3$·xEDAPbI_4/Spiro-OMeTAD/Ag	0.12	11.80%	Spin coat	150 °C
	2	2019	FTO/TiO_2/$CsPbI_3$/Spiro/Ag	0.1225<br>1.0	18.4%<br>16.1%	Spin coat	210 °C
	3	2019	ITO/PTAA/$CsPbI_3$/C60/BCP/Cu	0.16	12.50%	Thermal Evaporation	----
	4	2022	FTO/TiO_2/$CsPbI_3$/Spiro-MeOTAD/Au	1.0	16.80%	Spin coat	200 °C
	5	2022	ITO/PTAA/$CsPbI_3$/OMXene-$CsPbI_3$/CPTA/ BCP/Ag	25 cm^2<br>Minimodule	14.64%	Spray coat	150 °C
	6	2023	ITO/P3CT/$CsPbI_3$/DAB/PCBM/C60/BCP/Ag	1.0	16.71%	Blade coat	185 °C
	6	2023	FTO/TiO_2/SnO_2/$CsPbI_3$/Spiro/Au	26.77 cm^2<br>Minimodule	15.10%	Spin coat	185 °C
	7	2023	FTO/c-TiO_2/SnO_2/β-$CsPbI_3$/γ-$CsPbI_3$/$PC_{61}BM$/Ag	18.08 cm^2<br>Minimodule	18.43%	Spin coat + Evaporation	210 °C
	8	2023	FTO/c-TiO2/$CsPbI_3$ /spiro-OMeTAD/Au	12 cm^2<br>Minimodule	16.60%	Spin coat	190 °C
	9	2024	FTO/c-TiO_2/mp-TiO_2/ZrO_2/$CsPbI_3$:$EuCl_3$/Mesoporous C	1.44	4.97%	Drop cast	350°C
$CsPbI_xBr_{1-x}$	10	2017	FTO/c-TiO_2/$CsPbI_2Br$/P3HT/Au	0.159<br>1.2	7.7% ;<br>6.8%	Thermal Evaporation	300 °C
	11	2017	FTO/c-TiO_2/$Cs_{0.925}K_{0.075}PbBrI_2$/Spiro-OMeTAD/Au	0.15	10%	Spin coat	280 °C
	12	2018	FTO/TiO_2/$CsPbI_2Br$/Spiro-OMeTAD/Au	0.49<br>1.0	12.8%<br>11.6%	Spin coat	200 °C
	13	2018	FTO/c-TiO_2/$CsPbI_2Br$/ C	0.12	10.00%	Spin coat	340 °C
	14	2019	ITO/NiO/$CsPbI_2Br$/Nb_2O_5/Ag	0.155<br>5	14.45%<br>11.20%	Spin coat	160 °C
	15	2019	FTO/TiO_2/$CsPbI_2Br$/Spiro/Au	1.0	12.5%	Blade coat	150°C
	16	2019	FTO/c-TiO_2/mp-TiO_2/BaI_2:$CsPbI_2Br$/P3HT/Au	1.0	13.78%	Spin coat	280 °C
	17	2020	ITO/SnO_2/MgO/$CsPbBr_2I$/Spiro-OMeTAD/Ag	0.11	11.04%	Spin coat	160 °C
	18	2020	FTO/SnO_2/PANI doped $CsPbI_2Br$/ C	0.1	13.52%	Spin coat	280 °C
	19	2020	FTO/NiO/$InCl_3$:$CsPbI_2Br$/ZnO/C60+TPFPB+$LiClO_4$/Ag	1.0<br>10.92 cm^2<br>Minimodule	14.44%<br>12%	Spin coat	160 °C
	20	2021	FTO/bl-TiO_2/$CsPbBr_xI_{3-x}$/PTAA/Au	112 cm^2<br>Minimodule	13.82%	Spray coat	150 °C
	21	2021	ITO/ZnO/SnO_2/$CsPbI_2Br$/Spiro-OMeTAD/MoO_3/Ag	0.12	16.9%	Spin coat	180 °C
	22	2021	FTO/TiO_2/$InCl_3$:$CsPbI_2Br$/P3HT/Au	1.0	15.82%	Spin coat	280 °C
	23	2022	FTO/TiO_2/$GdCl_3$:$CsPbI_2Br$/Spiro-OMeTAD/Au	0.1	16.24%	Spin coat	160 °C
	24	2022	FTO/TiO_2/$CsPbI_{0.85}Br_{0.15}$/Spiro-OMeTAD/Au	1.0	17.21%	Spin coat	210 °C
	25	2022	FTO/TiO_2/$CsPb_{0.95}Tb_{0.05}I_2Br$/P3HT/Au	19.80 cm^2<br>Minimodule	10.94%	Spin coat	280 °C
$CsPbBr_3$	26	2018	FTO/c-TiO_2/$CsPbBr_3$/Spiro-MeOTAD/Au	1.0	5.37%	Thermal Evaporation	400~550 °C
	27	2018	FTO/TiO_2/$CsPbBr_3$/MoS_2 QDs/ C	1.0	4.12%	Spin coat + Spray coat	250 °C
	28	2018	FTO/c-TiO_2/m-TiO_2/$Cs_{1-x}Rb_xPbBr_3$/C	1.0	7.07%	Spin coat	250 °C
	29	2020	FTO/Ga-SnO_2/$CsPbBr_3$/ C	1.0	5.98%	Spin coat	250 °C
	30	2021	FTO/TiO_2/$CsPbBr_3$/ C	1.0	8.21%	Spin coat + Spray coat	250 °C
	31	2022	FTO/c-TiO_2/meso-TiO_2/$CsPbBr_3$/C	0.25<br>1.0	8.59%<br>7.81%	Inkjet print	150 °C

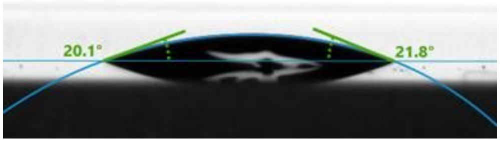

Figure S1. The water contact angle results of a pure glass surface.

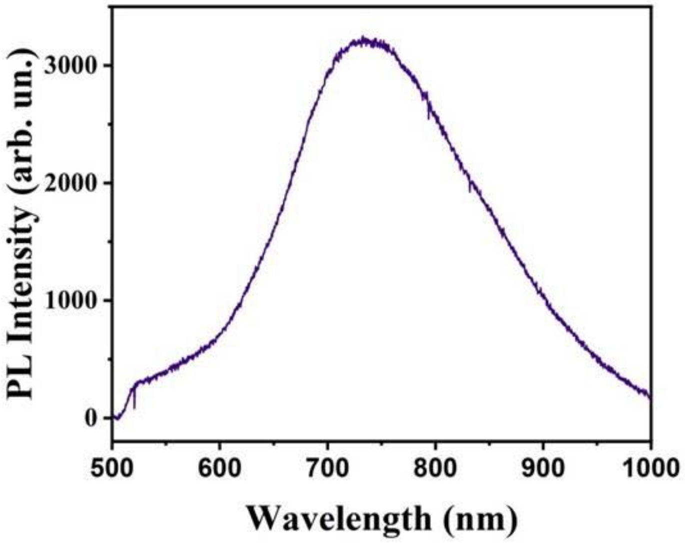

Figure S2. The photoluminescence (PL) results of $HPbI_3$ powder.

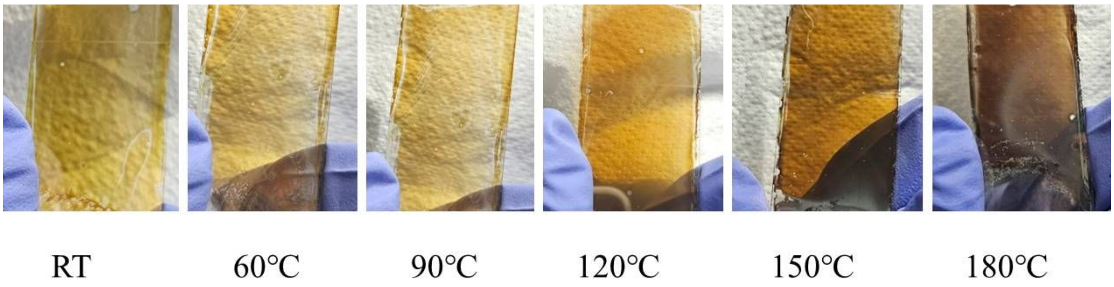

Figure S3. Photos of $CsPbI_3$ film samples obtained with IPA bath treatment and annealing for 10 minutes at room temperature (RT), 60°C, 90°C, 120°C, 150°C, and 180°C.

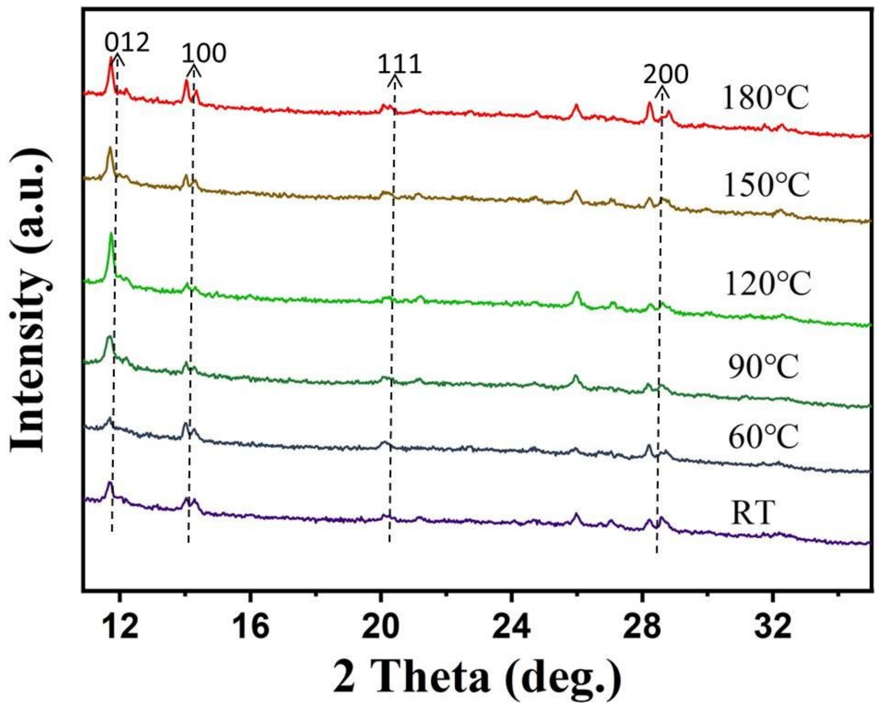

Figure S4. XRD results of $CsPbI_3$ samples with IPA bath treatment and annealed at different temperatures.

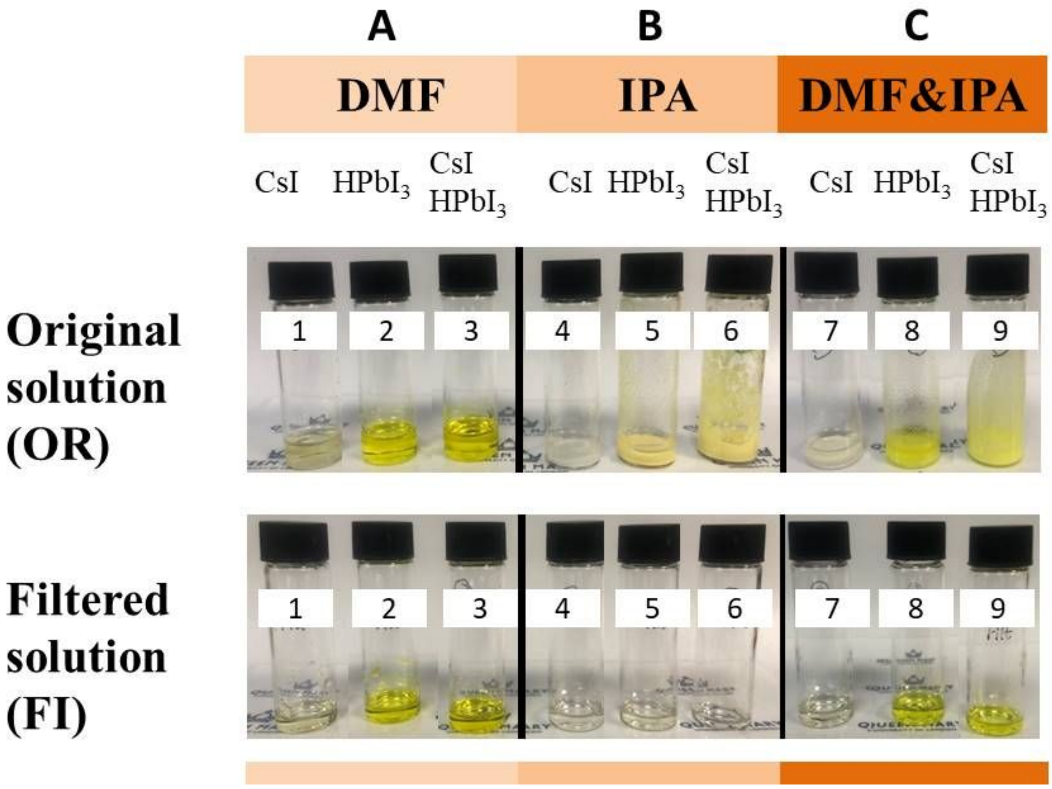

Figure S5. Solubility test visual photos. CsI, $HPbI_3$, and their mixtures dissolved in DMF, IPA, and a DMF and IPA mixed solution. The Original solution (OR) row refers to the liquid after shaking and stirring; The Filtered solution (FI) row refers to the clear liquid after filtration.

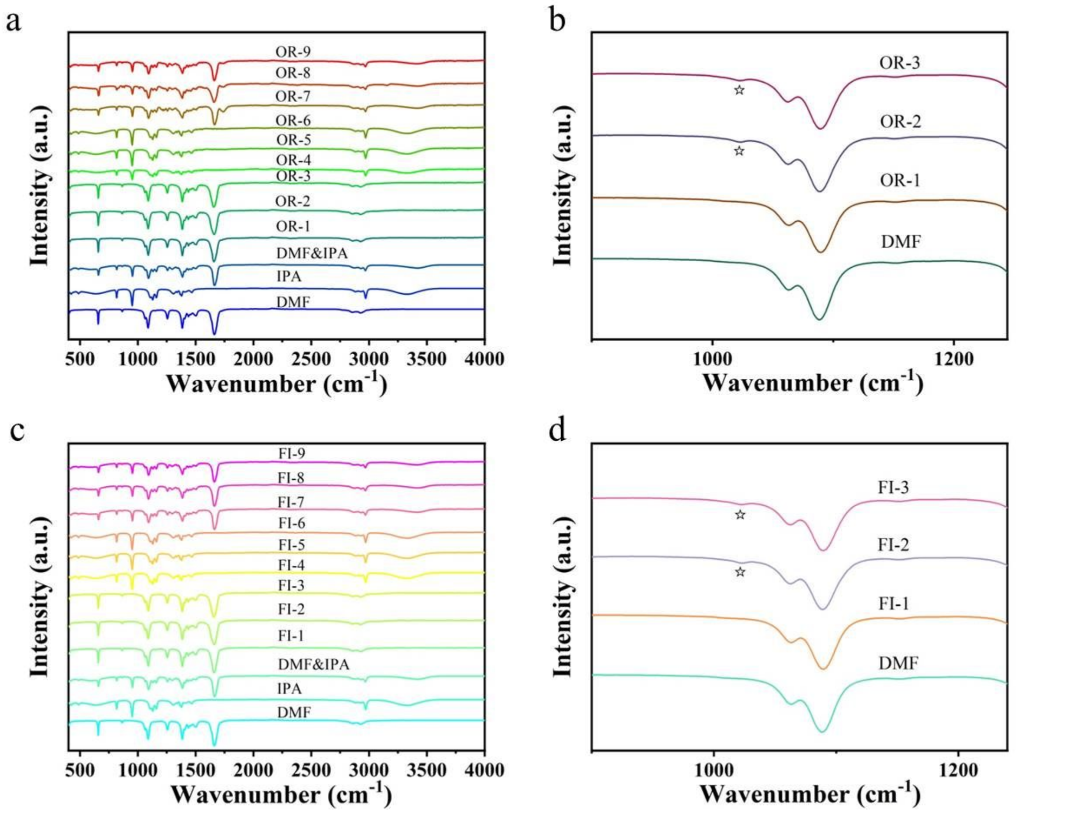

Figure S6. FTIR results in transmission mode: (a) All solutions of the OR series; (b) DMF solutions of the OR series; (c) All solutions of the FI series; (d) DMF solution of the FI series.

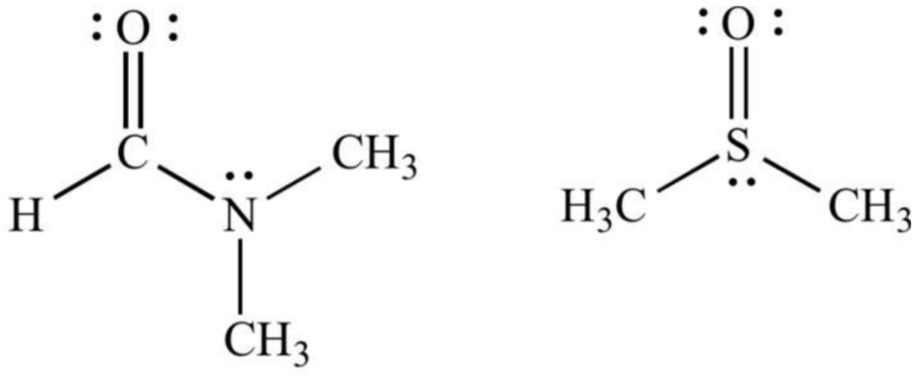

DMF **DMSO**

Figure S7. Chemical molecular structures of DMF and DMSO.

References

(1) Zhang, T.; Dar, M. I.; Li, G.; Xu, F.; Guo, N.; Grätzel, M.; Zhao, Y. Bication lead iodide 2D perovskite component to stabilize inorganic α-$CsPbI_3$ perovskite phase for high-efficiency solar cells. *Science advances* 2017, *3* (9), e1700841. DOI: doi:10.1126/sciadv.1700841.

(2) Wang, Y.; Dar, M. I.; Ono, L. K.; Zhang, T.; Kan, M.; Li, Y.; Zhang, L.; Wang, X.; Yang, Y.; Gao, X.; et al. Thermodynamically stabilized β-$CsPbI_3$–based perovskite solar cells with efficiencies >18%. *Science* 2019, *365* (6453), 591-595. DOI: doi:10.1126/science.aav8680.

(3) Becker, P.; Márquez, J. A.; Just, J.; Al-Ashouri, A.; Hages, C.; Hempel, H.; Jošt, M.; Albrecht, S.; Frahm, R.; Unold, T. Low Temperature Synthesis of Stable γ-$CsPbI_3$ Perovskite Layers for Solar Cells Obtained by High Throughput Experimentation. *Advanced Energy Materials* 2019, *9* (22), 1900555. DOI: https://doi.org/10.1002/aenm.201900555.

(4) Yu, G.; Jiang, K.-J.; Gu, W.-M.; Li, Y.; Lin, Y.; Xu, Y.; Jiao, X.; Xue, T.; Zhang, Y.; Song, Y. Vacuum-Assisted Thermal Annealing of $CsPbI_3$ for Highly Stable and Efficient Inorganic Perovskite Solar Cells. *Angewandte Chemie International Edition* 2022, *61* (27), e202203778. DOI: https://doi.org/10.1002/anie.202203778.

(5) Heo, J. H.; Zhang, F.; Park, J. K.; Joon Lee, H.; Lee, D. S.; Heo, S. J.; Luther, J. M.; Berry, J. J.; Zhu, K.; Im, S. H. Surface engineering with oxidized $Ti_3C_2T_x$ MXene enables efficient and stable p-i-n-structured CsPbI3 perovskite solar cells. *Joule* 2022, *6* (7), 1672-1688. DOI: https://doi.org/10.1016/j.joule.2022.05.013.

(6) Sun, N.; Fu, S.; Li, Y.; Chen, L.; Chung, J.; Saeed, M. M.; Dolia, K.; Rahimi, A.; Li, C.; Song, Z.; et al. Tailoring Crystallization Dynamics of $CsPbI_3$ for Scalable Production of Efficient Inorganic Perovskite Solar Cells. *Advanced Functional Materials* 2023, *34* (6), 2309894. DOI: https://doi.org/10.1002/adfm.202309894.

(7) Mali, S. S.; Patil, J. V.; Shao, J.-Y.; Zhong, Y.-W.; Rondiya, S. R.; Dzade, N. Y.; Hong, C. K. Phase-heterojunction all-inorganic perovskite solar cells surpassing 21.5% efficiency. *Nature Energy* 2023, *8* (9), 989-1001. DOI: 10.1038/s41560-023-01310-y.

(8) Tan, S.; Tan, C.; Cui, Y.; Yu, B.; Li, Y.; Wu, H.; Shi, J.; Luo, Y.; Li, D.; Meng, Q. Constructing an Interfacial Gradient Heterostructure Enables Efficient $CsPbI_3$ Perovskite Solar Cells and Printed Minimodules. *Advanced Materials* 2023, *35* (28), 2301879. DOI: https://doi.org/10.1002/adma.202301879.

(9) Valastro, S.; Calogero, G.; Smecca, E.; Bongiorno, C.; Arena, V.; Mannino, G.; Deretzis, I.; Fisicaro, G.; La Magna, A.; Alberti, A. Performance Evaluation of Printable Carbon-Based Perovskite Solar Cells Infiltrated with Reusable $CsPbI_3$:$EuCl_3$ and Standard AVA-$MAPbI_3$. *Solar RRL* 2024, *8* (5), 2300944. DOI: https://doi.org/10.1002/solr.202300944.

(10) Ma, Q.; Huang, S.; Chen, S.; Zhang, M.; Lau, C. F. J.; Lockrey, M. N.; Mulmudi, H. K.; Shan, Y.; Yao, J.; Zheng, J.; et al. The Effect of Stoichiometry on the Stability of Inorganic Cesium Lead Mixed-Halide Perovskites Solar Cells. *The Journal of Physical Chemistry C* 2017, *121* (36), 19642-19649. DOI: 10.1021/acs.jpcc.7b06268.

(11) Nam, J. K.; Chai, S. U.; Cha, W.; Choi, Y. J.; Kim, W.; Jung, M. S.; Kwon, J.; Kim, D.; Park, J. H. Potassium Incorporation for Enhanced Performance and Stability of Fully Inorganic Cesium Lead Halide Perovskite Solar Cells. *Nano Letters* 2017, *17* (3), 2028-2033. DOI: 10.1021/acs.nanolett.7b00050.

(12) Yin, G.; Zhao, H.; Jiang, H.; Yuan, S.; Niu, T.; Zhao, K.; Liu, Z.; Liu, S. Precursor Engineering for All-Inorganic $CsPbI_2Br$ Perovskite Solar Cells with 14.78% Efficiency. *Advanced*

Functional Materials 2018, *28* (39), 1803269. DOI: https://doi.org/10.1002/adfm.201803269.
(13) Dong, C.; Han, X.; Zhao, Y.; Li, J.; Chang, L.; Zhao, W. A Green Anti-Solvent Process for High Performance Carbon-Based $CsPbI_2Br$ All-Inorganic Perovskite Solar Cell. *Solar RRL* 2018, *2* (9), 1800139. DOI: https://doi.org/10.1002/solr.201800139.
(14) Liu, X.; Xiao, Y.; Zeng, Q.; Jiang, J.; Li, Y. Large-Area Organic-Free Perovskite Solar Cells with High Thermal Stability. *The journal of physical chemistry letters* 2019, *10* (20), 6382-6388. DOI: 10.1021/acs.jpclett.9b02644.
(15) Fan, Y.; Fang, J.; Chang, X.; Tang, M.-C.; Barrit, D.; Xu, Z.; Jiang, Z.; Wen, J.; Zhao, H.; Niu, T.; et al. Scalable Ambient Fabrication of High-Performance $CsPbI_2Br$ Solar Cells. *Joule* 2019, *3* (10), 2485-2502. DOI: https://doi.org/10.1016/j.joule.2019.07.015.
(16) Mali, S. S.; Patil, J. V.; Hong, C. K. Hot-Air-Assisted Fully Air-Processed Barium Incorporated $CsPbI_2Br$ Perovskite Thin Films for Highly Efficient and Stable All-Inorganic Perovskite Solar Cells. *Nano Letters* 2019, *19* (9), 6213-6220. DOI: 10.1021/acs.nanolett.9b02277.
(17) Wang, H.; Li, H.; Cao, S.; Wang, M.; Chen, J.; Zang, Z. Interface Modulator of Ultrathin Magnesium Oxide for Low-Temperature-Processed Inorganic $CsPbIBr_2$ Perovskite Solar Cells with Efficiency Over 11%. *Solar RRL* 2020, *4* (9), 2000226. DOI: https://doi.org/10.1002/solr.202000226.
(18) Liu, C.; He, J.; Wu, M.; Wu, Y.; Du, P.; Fan, L.; Zhang, Q.; Wang, D.; Zhang, T. All-Inorganic $CsPbI_2Br$ Perovskite Solar Cell with Open-Circuit Voltage over 1.3 V by Balancing Electron and Hole Transport. *Solar RRL* 2020, *4* (7), 2000016. DOI: https://doi.org/10.1002/solr.202000016.
(19) Liu, C.; Yang, Y.; Zhang, C.; Wu, S.; Wei, L.; Guo, F.; Arumugam, G. M.; Hu, J.; Liu, X.; Lin, J.; et al. Tailoring C60 for Efficient Inorganic $CsPbI_2Br$ Perovskite Solar Cells and Modules. *Advanced Materials* 2020, *32* (8), 1907361. DOI: https://doi.org/10.1002/adma.201907361.
(20) Heo, J. H.; Zhang, F.; Xiao, C.; Heo, S. J.; Park, J. K.; Berry, J. J.; Zhu, K.; Im, S. H. Efficient and Stable Graded $CsPbI_{3-x}Br_x$ Perovskite Solar Cells and Submodules by Orthogonal Processable Spray Coating. *Joule* 2021, *5* (2), 481-494. DOI: https://doi.org/10.1016/j.joule.2020.12.010.
(21) Zhou, D.; Huang, J.; Liu, J.; Yan, H.; Zhang, J.; Zhang, M.; Liang, G.; Lu, L.; Zhang, X.; Xu, P.; et al. Dual Passivation Strategy for High Efficiency Inorganic $CsPbI_2Br$ Solar Cells. *Solar RRL* 2021, *5* (5), 2100112. DOI: https://doi.org/10.1002/solr.202100112.
(22) Mali, S. S.; Patil, J. V.; Shinde, P. S.; de Miguel, G.; Hong, C. K. Fully Air-Processed Dynamic Hot-Air-Assisted M:$CsPbI_2Br$ (M: Eu^{2+}, In^{3+}) for Stable Inorganic Perovskite Solar Cells. *Matter* 2021, *4* (2), 635-653. DOI: https://doi.org/10.1016/j.matt.2020.11.008.
(23) Pu, X.; Yang, J.; Wang, T.; Cheng, S.; Cao, Q.; Zhao, J.; Chen, H.; Zhang, Y.; Xu, T.; Tojiboyev, I.; et al. Gadolinium-incorporated $CsPbI_2Br$ for boosting efficiency and long-term stability of all-inorganic perovskite solar cells. *Journal of Energy Chemistry* 2022, *70*, 9-17. DOI: https://doi.org/10.1016/j.jechem.2022.02.004.
(24) Zhang, H.; Xiang, W.; Zuo, X.; Gu, X.; Zhang, S.; Du, Y.; Wang, Z.; Liu, Y.; Wu, H.; Wang, P.; et al. Fluorine-Containing Passivation Layer via Surface Chelation for Inorganic Perovskite Solar Cells. *Angewandte Chemie International Edition* 2023, *62* (6), e202216634. DOI: https://doi.org/10.1002/anie.202216634.
(25) Mali, S. S.; Patil, J. V.; Rondiya, S. R.; Dzade, N. Y.; Steele, J. A.; Nazeeruddin, M. K.; Patil,

P. S.; Hong, C. K. Terbium-Doped and Dual-Passivated γ-$CsPb(I_{1-x}Br_x)_3$ Inorganic Perovskite Solar Cells with Improved Air Thermal Stability and High Efficiency. *Advanced Materials* 2022, *34* (29), 2203204. DOI: https://doi.org/10.1002/adma.202203204.
(26) Lei, J.; Gao, F.; Wang, H.; Li, J.; Jiang, J.; Wu, X.; Gao, R.; Yang, Z.; Liu, S. Efficient planar $CsPbBr_3$ perovskite solar cells by dual-source vacuum evaporation. *Solar Energy Materials and Solar Cells* 2018, *187*, 1-8. DOI: https://doi.org/10.1016/j.solmat.2018.07.009.
(27) Duan, J.; Dou, D.; Zhao, Y.; Wang, Y.; Yang, X.; Yuan, H.; He, B.; Tang, Q. Spray-assisted deposition of $CsPbBr_3$ films in ambient air for large-area inorganic perovskite solar cells. *Materials Today Energy* 2018, *10*, 146-152. DOI: https://doi.org/10.1016/j.mtener.2018.09.001.
(28) Li, Y.; Duan, J.; Yuan, H.; Zhao, Y.; He, B.; Tang, Q. Lattice Modulation of Alkali Metal Cations Doped $Cs_{1-x}R_xPbBr_3$ Halides for Inorganic Perovskite Solar Cells. *Solar RRL* 2018, *2* (10), 1800164. DOI: https://doi.org/10.1002/solr.201800164.
(29) Zhao, Y.; Deng, Q.; Guo, R.; Wu, Z.; Li, Y.; Duan, Y.; Shen, Y.; Zhang, W.; Shao, G. Sputtered Ga-Doped SnO_x Electron Transport Layer for Large-Area All-Inorganic Perovskite Solar Cells. *ACS applied materials & interfaces* 2020, *12* (49), 54904-54915. DOI: 10.1021/acsami.0c19540.
(30) Zhang, Z.; Ba, Y.; Chen, D.; Ma, J.; Zhu, W.; Xi, H.; Chen, D.; Zhang, J.; Zhang, C.; Hao, Y. Generic water-based spray-assisted growth for scalable high-efficiency carbon-electrode all-inorganic perovskite solar cells. *iScience* 2021, *24* (11), 103365. DOI: https://doi.org/10.1016/j.isci.2021.103365.
(31) Zhang, L.; Chen, S.; Zeng, J.; Jiang, Z.; Ai, Q.; Zhang, X.; Hu, B.; Wang, X.; Yang, S.; Xu, B. Inkjet-Printing Controlled Phase Evolution Boosts the Efficiency of Hole Transport Material Free and Carbon-Based $CsPbBr_3$ Perovskite Solar Cells Exceeding 9%. *Energy & Environmental Materials* 2022, *7* (2), e12543. DOI: https://doi.org/10.1002/eem2.12543.
